# What Causes Specific Language Impairment in Children?

**DOI:** 10.1111/j.1467-8721.2006.00439.x

**Published:** 2006-10

**Authors:** Dorothy V M Bishop

**Affiliations:** University of Oxford Oxford, England

**Keywords:** genetics, specific language impairment, twins, etiology

## Abstract

Specific language impairment (SLI) is diagnosed when a child's language development is deficient for no obvious reason. For many years, there was a tendency to assume that SLI was caused by factors such as poor parenting, subtle brain damage around the time of birth, or transient hearing loss. Subsequently it became clear that these factors were far less important than genes in determining risk for SLI. A quest to find “the gene for SLI” was undertaken, but it soon became apparent that no single cause could account for all cases. Furthermore, although fascinating cases of SLI caused by a single mutation have been discovered, in most children the disorder has a more complex basis, with several genetic and environmental risk factors interacting. The clearest evidence for genetic effects has come from studies that diagnosed SLI using theoretically motivated measures of underlying cognitive deficits rather than conventional clinical criteria.

Talking comes so naturally to most children that one seldom pauses to consider the enormous complexity of the achievement. Understanding just how the human brain manages to learn language—typically in the space of around 4 short years—is still a long way off. Perhaps as remarkable as the speed with which young humans learn language is the robustness of this process in the face of adverse conditions (Bishop & Mogford, 1993). Most children will learn to talk adequately even if they are exposed to impoverished language input from adults or are visually impaired and thus unable to see what is being talked about. Children who are unable to speak because of physical disability, and those who cannot hear what others say to them, will nevertheless learn to communicate by other means, provided they are exposed to alternative systems of communication such as sign language.

There are, however, exceptions to this general rule of speedy and robust language acquisition: Children with specific language impairment (SLI) have major problems in learning to talk, despite showing normal development in all other areas (see Table 1). Thus, a typical 7- or 8-year-old child with SLI may talk like a 3-year-old, using simplified speech sounds, with words strung together in short, ungrammatical strings—e.g., “me go there,” rather than “I went there.” SLI is a heterogeneous category, varying in both severity and profile of disorder, but in most cases it is possible to demonstrate problems with both understanding and producing spoken language; for example, the child may have difficulty using toys to act out a sentence such as “the boy is chased by the dog,” showing confusion as to who is doing what to whom. Language impairment in SLI is puzzling precisely because it occurs in children who are otherwise normally developing, with no hearing problems or physical handicaps that could explain the difficulties.

**TABLE 1:** Characteristics of Specific Language Impairment (SLI)

Diagnostic criteria
• Language is significantly below level expected from age and IQ, usually interpreted as scoring in the lowest 10% on a standardized test of expressive and/or receptive language
• Nonverbal IQ and nonlinguistic aspects of development (self-help skills, social skills) fall within broadly normal limits
• Language difficulties cannot be accounted for by hearing loss, physical abnormality of the speech apparatus, or environmental deprivation
• Language difficulties are not caused by brain damage
Common presenting features*
• Delay in starting to talk; first words may not appear until 2 years of age or later
• Immature or deviant production of speech sounds, especially in preschool children
• Use of simplified grammatical structures, such as omission of past tense endings or the auxiliary “is,” well beyond the age when this is usually mastered
• Restricted vocabulary, in both production and comprehension
• Weak verbal short-term memory, as evidenced in tasks requiring repetition of words or sentences
• Difficulties in understanding complex language, especially when the speaker talks rapidly

* SLI shows substantial heterogeneity, as well as age-related change, and diagnosis does not depend on presence or absence of specific language characteristics.

The prevalence of SLI has been estimated at around 7% (Tomblin et al., 1997), although this will vary with both the diagnostic criteria and children's age: Long-term language impairments that persist into adulthood are less common than milder delays in preschoolers, which may resolve with time (Bishop & Adams, 1990).

## SLI AS A STRONGLY GENETIC DISORDER

When I started out studying SLI in the mid-1970s, very little was known about its causes. Possibilities that had been suggested included inadequate parenting, subtle brain damage acquired around the time of birth, or recurrent ear disease in early childhood. However, none of these theories has had much support. Instead, it has become increasingly clear that genetic makeup exerts a strong influence in determining which children will develop SLI. Studies showing that SLI tends to run in families are suggestive of genetic influence, but they are not watertight, because family members share environments as well as genes. More compelling evidence comes from twin studies showing that monozygotic (MZ) twins, who are genetically identical, resemble each other in terms of SLI diagnosis more closely than do dizygotic (DZ) twins, who have 50% of their segregating genes (genes that can take different forms, or alleles, in different people) in common. Statistical analysis of twin data shows that the environment shared by the twins is relatively unimportant in causing SLI, whereas genes exert a significant effect, with heritability estimates (i.e., the proportion of variance in a trait that is attributable to genetic factors) typically ranging from around .5 to .75 for school-aged children (see Bishop, 2002, for review).

## IS THERE A “GENE FOR LANGUAGE”?

When it first became apparent that genes are implicated in SLI, there was a lot of popular interest in the idea that researchers might discover a “gene for language” that had evolved in humans and that distinguished humans from other primates. The idea would be that this gene was defective in some children, who consequently lacked a natural capacity for language learning. However, subsequent research on SLI has not supported this interpretation. For one thing, it is unusual to find families in which SLI is inherited in a simple fashion. In this regard, SLI resembles *complex genetic disorders*, such as asthma and diabetes, which run in families but for which patterns of inheritance do not correspond to any known dominant or recessive pattern.

There is, however, one remarkable family that is an exception: the three-generational KE family from London, England, that has been extensively studied by geneticists. SLI affects 50% of the children of an affected parent, and it is caused by a mutation affecting a tiny piece of DNA on a gene on chromosome 7. The KE family excited a great deal of interest from researchers, because, once the defective gene was identified, it was possible to study its effect on the developing brain. However, research on this gene, known as *FOXP2*, makes it clear that it is not a gene for language—rather, it is a gene that regulates the activity of other genes, having an effect on the development of many organs, including brain systems important for speech and language (Fisher, 2005). Structural and functional brain-imaging studies have shown that affected family members have abnormalities in the caudate nuclei and cerebellum as well as in Broca's area, a classic language center (Vargha-Khadem, Gadian, Copp, & Mishkin, 2005). Studies of the KE family have helped to identify one route by which genetic variation affects brain development and subsequent language capability, but it is clear that this is only part of the picture. Most people with SLI do not have any abnormality of the *FOXP2* gene, and it seems likely that in the majority of cases the disorder is caused by the interaction of several genes together with environmental risk factors.

## GENETIC INFLUENCES ON DIFFERENT ASPECTS OF LANGUAGE IMPAIRMENT

The first step in unravelling the causes of a condition such as SLI does not involve any direct DNA analysis, but rather uses methods such as twin studies, which allow the comparison of phenotypes (observed characteristics) in people who differ in their degree of genetic similarity. One issue is how to define the SLI phenotype. In one of the first twin studies that I did on this topic, I found that it was very common to find MZ twin pairs in which one twin met clinical criteria for SLI and the other did not. However, the non-SLI twin typically had evidence of language difficulties: These simply were not selective enough or persistent enough to meet conventional diagnostic criteria for the disorder. This suggested that simply categorising children as affected or unaffected on the basis of conventional language tests was not an effective approach to phenotype definition. An alternative approach is to look for *endophenotypes*, measures of underlying factors thought to play a causal role in the disorder (Gottesman & Gould, 2003). I adopted such an approach by doing genetic analysis using experimental measures that were derived from particular theoretical accounts of SLI.

**Fig. 1:**
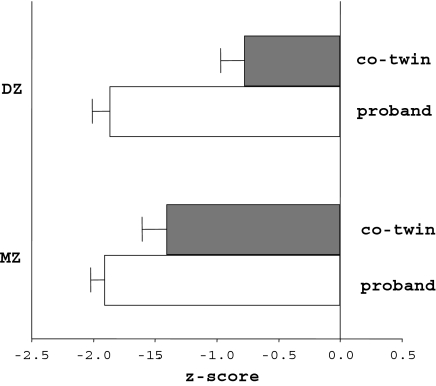
Mean z-scores on nonword repetition for individuals with specific language impairment (probands, defined as those with z-score less than 1.0) and their co-twins, in relation to whether they are monozygotic (MZ) or dizygotic (DZ) twins. The population mean score is zero. Insofar as similar environmental influences affect both twins, two members of a twin pair would be expected to resemble one another. However, if, as shown here, the similarity between MZ probands and their co-twins is greater than that between DZ probands and their co-twins, this points to a genetic influence on low scores. Data from Bishop, North, & Donlan (1996).

One such measure, nonword repetition, was derived from a theory that attributes SLI to impairment in a system that is specialised for holding verbal material in memory for short periods of time—phonological short-term memory (STM). An estimate of phonological STM capacity can be obtained by asking children to repeat meaningless sequences of syllables, such as “perplisteronk” or “blonterstaping.” Children with SLI are usually extremely poor at this task, even if they can produce the individual speech sounds accurately. The longer the nonsense word, the worse they do. The task also reveals deficits in people who appear to have overcome early developmental language difficulties, and so it acts as a good marker of resolved language difficulties. When we used this task in a twin study (Bishop, North, & Donlan, 1996), we found evidence of strong genetic influence on impaired nonword repetition (see Fig. 1). Subsequently, molecular geneticists have homed in on an area of chromosome 16 that appears to harbor a gene associated with poor phonological STM (Newbury, Bishop, & Monaco, 2005).

We know that phonological STM is poor in SLI, but there has been debate as to whether this can be traced to a more general deficit affecting perception of auditory input. One account proposes that the fundamental problem in SLI is a difficulty in distinguishing or identifying sounds that are brief or that occur in rapid succession, be they nonverbal or verbal. Accordingly, in one twin study we included a measure of nonverbal auditory perception (identification of tone sequences), as well as a test of nonword repetition. Although we found evidence for poor performance in SLI on both tasks, the twin analysis suggested they were not different manifestations of the same problem. Deficient nonword repetition again showed strong genetic influence, but poor ability to identify tone sequences was not significantly heritable. Twins tended to resemble one another on the nonverbal auditory task, but this was equally true for DZ as for MZ twins, suggesting the twin similarity was the result of environmental influences that they shared. One possibility is that this task is affected by the child's musical experiences: I showed that the effect of shared environment on the tone-sequence task accounted for about 60% of the variance, but almost half of this effect could be accounted for by a measure of the amount of live music experienced at home (as assessed by a parental questionnaire asking whether family members played a musical instrument; Bishop, 2001).

In a recent study with a sample of 6-year-old twins, we again measured phonological STM, but this time we also took a measure of children's ability to add appropriate inflectional endings to verbs (Bishop, Adams, & Norbury, 2006). Many English-speaking children with SLI have unusual difficulty with some aspects of grammar, and will tend to omit the appropriate verb inflection in sentences such as “Yesterday my brother walk(ed) to school,” or “Every day John ride(s) his bike.” There has been debate in the field as to whether this grammatical difficulty is a consequence of weak phonological STM or has separate origins. We found evidence for strong genetic influence on poor performance on the verb-inflection task: If a MZ twin had a low score, his or her co-twin also tended to do poorly, whereas if a DZ twin did poorly, the result for the co-twin was much more variable. However, there was no association between this effect and that seen on phonological STM, where again significant heritability of the deficit was seen. Both impairments were found in SLI, and both were heritable, yet they were only weakly correlated, and genetic analysis suggested that different genes were implicated in the two deficits.

## SLI AS A DISORDER OF MULTIPLE UNDERLYING DEFICITS

As argued in the previous section, various underlying skills are impaired in SLI, but these different deficits have different causes, some genetic and some environmental. Our first reaction to such results was to think that the genetic analysis might help us identify distinct subgroups of SLI, each with a different underlying cause. However, what repeatedly emerged in our studies was that children who had a single area of deficit were less likely to be identified clinically as cases of SLI than were those who had more than one deficit. Thus, although different deficits have different origins and can be dissociated, it seems as though a child has to be impaired in more than one domain in order for language to be seriously impaired. This brings us back to the point made at the start of this article: Language is usually surprisingly robust in the face of adverse developmental circumstances. This suggests that there may be multiple routes to effective language acquisition, and if one route is blocked, another can usually be found. However, if two or more routes are blocked, then language learning will be compromised. Many researchers are still engaged in the quest for a parsimonious single-factor theory of SLI. However, the genetic studies are forcing us to rethink this perspective and to regard SLI as a case in which development is compromised precisely because more than one cognitive process is disrupted (Bishop, 2006). This conceptualisation challenges any notion of SLI as a single syndrome and also suggests that we may need to analyze it in terms of dimensions of impairment instead of looking for discrete subtypes.

## CLINICAL IMPLICATIONS

All too often people assume that genes exert a deterministic effect and that nothing can be done to help a child whose impairment has a constitutional origin. This is a serious misconception. To say that a disorder is highly heritable is to imply that variations in children's genetic makeup are more important than variations in their environmental experiences in determining who has a disorder. However, it says nothing about how the child might respond to a novel intervention that is not usually encountered in the environment. By analogy, consider the case of Huntington's disease, a progressive late-onset degenerative disease that is caused by a dominantly inherited mutation. Mouse models have shown that onset and severity of the motor symptoms can be modified by early-environmental enrichment (Spires & Hannan, 2005). So even in the case of a strongly genetic disorder, environmental modifications can have an effect. And in a disorder such as SLI, in which multiple genetic and environmental risk factors are implicated, there is every reason to suppose that ways of modifying the course of the disorder may be discovered, especially if new genetic knowledge is used to identify children at risk early so that intervention can begin at a young age.

## FUTURE DIRECTIONS

The study of SLI is a field in which interdisciplinary collaboration is vital. It is sometimes assumed that once a disorder is discovered to have a genetic component, the psychologists have no further role to play, and the only task left is for molecular geneticists to isolate the gene responsible. SLI provides a clear counterexample to such reasoning, demonstrating that without theoretically motivated measures of the underlying phenotype, geneticists are unlikely to make progress in unravelling the causes of these complex but common developmental disorders. The task for psychologists is to identify which components of language show significant heritability and, hence, constitute good candidates for genetic analysis, as well as to discover new endophenotypes. Measures of phonological STM and use of grammatical morphology have already been discussed as showing good potential in this regard. A further promising approach would be to use dynamic measures that assess how well children respond to particular kinds of interventions—i.e., measure the extent to which language abilities can be modified, rather than taking a single measure at one point in time (e.g., Peña, Iglesias, & Lidz, 2001).
